# Splenic Stromal Cells from Aged Mice Produce Higher Levels of IL-6 Compared to Young Mice

**DOI:** 10.1155/2014/826987

**Published:** 2014-03-04

**Authors:** Jihyun Park, Takuya Miyakawa, Aya Shiokawa, Haruyo Nakajima-Adachi, Masaru Tanokura, Satoshi Hachimura

**Affiliations:** ^1^Research Center for Food Safety, Graduate School of Agricultural and Life Sciences, The University of Tokyo, 1-1-1 Yayoi, Bunkyo-ku, Tokyo 113-8657, Japan; ^2^Department of Applied Biological Chemistry, Graduate School of Agricultural and Life Sciences, The University of Tokyo, 1-1-1 Yayoi, Bunkyo-ku, Tokyo 113-8657, Japan

## Abstract

Inflamm-aging indicates the chronic inflammatory state resulting from increased secretion of proinflammatory cytokines and mediators such as IL-6 in the elderly. Our principle objective was to identify cell types that were affected with aging concerning IL-6 secretion in the murine model. We compared IL-6 production in spleen cells from both young and aged mice and isolated several types of cells from spleen and investigated IL-6 mRNA expression and protein production. IL-6 protein productions in cultured stromal cells from aged mice spleen were significantly high compared to young mice upon LPS stimulation. IL-6 mRNA expression level of freshly isolated stromal cells from aged mice was high compared to young mice. Furthermore, stromal cells of aged mice highly expressed IL-6 mRNA after LPS injection in vivo. These results suggest that stromal cells play a role in producing IL-6 in aged mice and imply that they contribute to the chronic inflammatory condition in the elderly.

## 1. Introduction

The elderly show differences in their ability to resist disease or injury compared to the young and adults, and some of the noticeable features are alterations in the development and maintenance of immune response and cellular function [1–3]. Aging process triggers suchlike changes of immune functions and as a result of dysregulated immune functions against infection and inflammation, a number of complications and chronic medical conditions occur [3–5].

Systemic inflammatory response syndrome is induced by dysregulated immune response to trauma or infection that normally protective responses became harmful provoking shock and organ dysfunctions [3]. Inflamm-aging indicates the chronic inflammatory state resulting from increased secretion of proinflammatory cytokines and mediators in the elderly [6, 7]. Partly due to inflamm-aging, the elderly are susceptible to autoimmune diseases, bacterial pathogens, and viral exposure, and the occurrence of autoimmune diseases, infectious diseases, and cancer increases in the elderly [8, 9]. Also a number of immune alterations, such as changed cytokine profile, might facilitate allergic associated conditions in the elderly [10, 11].

Proinflammatory cytokines, such as TNF-*α*, IL-1, and IL-6, are significantly related to the age-associated diseases and disability, and these are important factors on inflammatory response in the elderly [12–14]. Especially, IL-6 is considered as a dependable aging parameter [15]. Aged IL-6 knockout mice show reduced mortality compared to wild type mice after injection with LPS and anti-IL-6 receptor antibodies are good formulation for rheumatoid arthritis [3]. IL-6 produced in multiple tissues during inflammation stimulates immune process, and the production of IL-6 is elevated in some tissues of aged mice compared to young adult mice such as lung, kidney, skeletal muscle, heart, fat, and spleen [3, 16]. And under stressful or depressed conditions, IL-6 levels are increased [17]. Moreover, IL-6 is detected in high levels in disease conditions including autoimmune disease, such as rheumatoid arthritis, atherosclerosis, diabetes, and inflammatory-bowel diseases [18, 19]. It implies that IL-6 might play roles that boost the risk and mortality in inflammation in the elderly.

IL-6 is mainly produced by cells such as macrophages and dendritic cells. In secondary lymphoid organs (SLOs), where immune responses are initiated, a number of hematopoietic cells, including macrophages and dendritic cells which were mentioned above, interact with each other [20]. In addition to these immune cells, stromal cells are also present in SLOs. Stromal cells have been considered as cells that do not significantly affect the immune response but give a structural support to the lymphoid organs; however, the functions of stromal cells in immune homeostasis and immune responses have begun to emerge [21].

In this study, our principle objective was to identify cell types that were affected by aging concerning secretion of IL-6 in the murine model. To answer these questions, we compared IL-6 production in splenocytes from both young and aged mice and isolated a number of cell types from spleen and investigated IL-6 mRNA or protein expression. We found that stromal cells express IL-6 highly with aging both in noninflammatory and inflammatory conditions.

## 2. Materials and Methods

### 2.1. Animals

C57BL/6 and BALB/c female mice were obtained from CLEA Japan (Tokyo, Japan). The mice were used in the experiments at 2–4 mo for young mice and at 11–18 mo for aged mice, respectively. All procedures were performed in accordance with the guidelines for animal use and care of the University of Tokyo.

### 2.2. Culture Medium

Cells were cultured in RPMI 1640 medium supplemented with 5% FCS, 100 U/mL penicillin, 100 *μ*g/mL streptomycin, 3 mM L-glutamine, and 50 *μ*M 2-mercaptoethanol.

### 2.3. Isolation of Cells and Cell Culture

Spleens were removed from mice and digested with collagenase type 1. After red blood cell lysis, white blood cells were counted. Five × 10^5^ whole splenocytes were seeded in 96-well plates in 200 *μ*L RPMI medium and incubated for 24 hours at 37°C and 5% CO_2_. Macrophages, CD11c^+^ dendritic cells, and CD45^−^ stromal cells were isolated by MACS (Miltenyi Biotech, Bergisch-Gladbach, Germany). In short, cells were incubated with anti-mouse F4/80, CD11b, CD11c, and CD45^−^ coated magnetic beads (Miltenyi Biotech), respectively, and selected on MACS separation columns (Miltenyi Biotech). Macrophages were prepared by F4/80 positive selection or CD11c negative selection and CD11b positive selection. Dendritic cells were isolated as CD11c^+^ cells and stromal cells as CD45^−^ cells, respectively. In certain experiments, CD45^+^ cells were also used. MACS purified macrophages, dendritic cells, or CD45^+^ cells were seeded in 96-well plates (5 × 10^5^ cells/well) in 200 *μ*L RPMI medium with or without LPS and cultured for 24 h. Five × 10^4^ cells of CD45^−^ stromal cells were cultured in the same manner as above. Since the splenic CD45^−^ stromal cells form a dense network and FRCs, a kind of stromal cells, are connected to each other in spleen forming a three-dimensional reticulum, lower concentrations of cells were used for measurement of IL-6 protein production compared to other cell types [22, 23].

### 2.4. Exposure to LPS

Acute inflammation was induced by i.p. injection with LPS derived from *E. coli* (Sigma-Aldrich, St. Louis, MO, USA). LPS was dissolved in phosphate-buffered saline (PBS) and administered by i.p. with a dose of 3.3 *μ*g/g body weight in 100 *μ*L in in vivo experiments. In control mice, PBS was injected. Ninety minutes after LPS injection, mice were sacrificed and spleens were aseptically removed. For in vitro experiments, LPS dissolved in distilled water was diluted to 1 *μ*g/mL and 10 *μ*g/mL with RPMI medium. Cells were cultured with LPS for 24 h and culture supernatants were subjected to ELISA.

### 2.5. Measurement of IL-6

IL-6 level in the culture supernatant was assayed by enzyme-linked immunosorbent assay (ELISA). Cells were cultured with medium containing 0, 1, or 10 *μ*g/mL LPS. Supernatants were collected after 24 h and analyzed by ELISA.

### 2.6. Quantitative PCR

Total RNA was isolated from purified cells by QIA shredder and RNeasy Mini kit (QIAGEN, Germantown, MD, USA) and by SV total RNA isolation system (Promega, Madison, WI, USA) from organs. Quantitative PCR reaction was performed with SYBR green PCR master mix (QIAGEN). Gene expression levels for each sample were normalized to GAPDH serving as internal standard. Sequences of the forward and reverse primers were as follows: GAPDH (*Gapdh*), 5′-TGTCCGTCGTGGATCTGAC-3′ and 5′-CCTGCTTCACCACCTTCTTG-3′; IL-6 *(Il6),* 5′-TGGAGTCACAGAAGGAGTGGCTAAG-3′ and 5′-TCTGACCACAGTGAGGAATGTCAA-3′; IL-17A *(Il17a)*, 5′-GAAGCTCAGTGCCGCCA-3′ and 5′-TTCATGTGGTGGTCCAGCTTT-3′; IL-1*β(Il1b)*, 5′-CAGGATGAGGACATGAGCAC-3′ and 5′-CAGTTGTCTAATGGGAACGTCA-3′.

### 2.7. Cell Population with Flow Cytometry Analysis

Spleens were recovered from young and aged mice and spleen cells were used for flow cytometry analysis after red blood cell lysis. Cell staining for flow cytometry was performed at 4°C after Fc-block step for 15 min with the following monoclonal antibodies: FITC-conjugated anti-CD11b, FITC-conjugated anti-CD4, FITC-conjugated anti-CD45, PE-conjugated anti-F4/80, PE-conjugated anti-gp38, biotinylated anti-CD31, and PE-conjugated anti-B220 for 20 min. All data were analyzed with FlowJo.

### 2.8. Statistical Analysis

Results comparisons were performed by Student's *t*-test. A value of *P* < 0.05 was considered to be significant.

## 3. Results

### 3.1. IL-6 Production of Spleen Cells Is Higher in Aged Mice Compared to Young Mice

**Figure 1:**
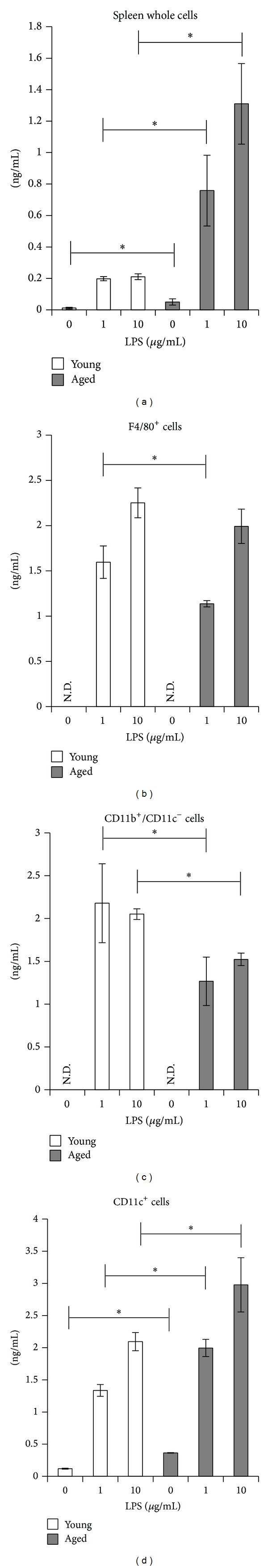
IL-6 protein production in splenocytes and their subpopulations from young and aged mice (*n* = 2~4). Whole splenocytes (a), spleen-derived F4/80^+^ cells (b), CD11b^+^/CD11c^−^ cells (c), and CD11c^+^ cells (d) from young and aged mice were cultured with 0, 1, and 10 *μ*g/mL LPS for 24 hours. Supernatants were collected from the cell culture and IL-6 was measured by ELISA. The results are expressed as mean ± SD. The data are the representative of two similar experiments. *P < 0.05.

To examine the aging effect on IL-6 protein production levels in the spleen of young and aged mice in vitro, splenocytes of young and aged mice were incubated with or without LPS for 24 h. After incubation, we analyzed IL-6 protein concentrations in supernatant by ELISA. IL-6 protein concentrations increased in the supernatant dependent on LPS concentration in both young and aged mice splenocytes (Figure 1(a)). In the spleen cells of aged mice, stimulation of LPS led to IL-6 protein upregulation in the supernatant, while in young mice splenocytes, although the concentration of IL-6 protein increased when stimulated with LPS, this was only a modest increase. The difference of IL-6 protein levels between young and aged mice became significantly greater in LPS-concentration dependent manner. Similar results were obtained not only for C57BL/6 mice but also for BALB/c mice (data not shown). Macrophages are well known for important cells that produce the major proinflammatory cytokines such as IL-6 and TNF-*α* [24]. To analyze the effect of the aging process on the IL-6 secretion of splenic macrophages, macrophages were isolated from spleen using two different cell markers. F4/80^+^ cells and CD11b^+^CD11c^−^ cells were prepared by using MACS. In either case, macrophages produced higher levels of IL-6 protein in young mice group than aged mice group after LPS stimulation (Figures 1(b) and 1(c)). Dendritic cells are also known as a source of IL-6 [25]. Dendritic cells from young and aged mice were isolated and IL-6 protein levels were investigated. Dendritic cells from the aged mice group produced slightly higher amounts of IL-6 proteins compared to young mice dendritic cells (Figure 1(d)). In case of the expression of IL-6 mRNA, however, the differences between young and aged mice were negligible (data not shown). Our results show that aging process does not affect the function of macrophages and dendritic cells in IL-6 secretion.

### 3.2. Stromal Cells from Aged Mice Produce Higher Levels of IL-6 Compared to Those from Young Mice

**Figure 2:**
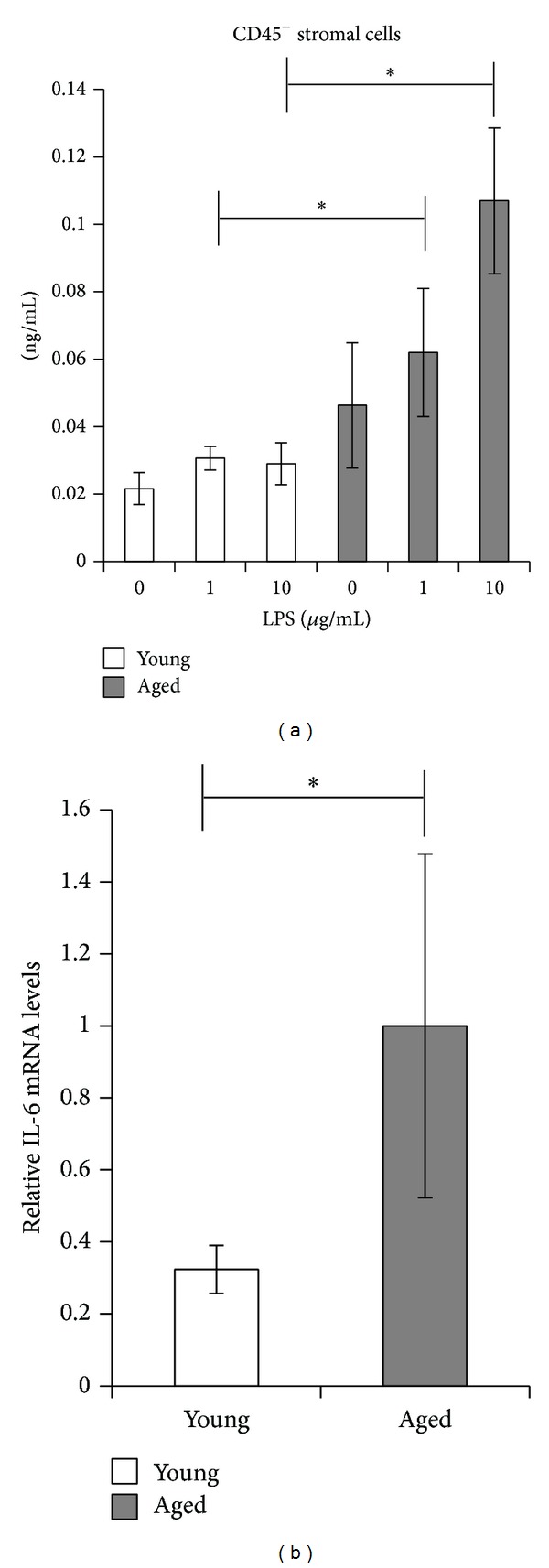
IL-6 protein induced by LPS in CD45^−^ stromal cells and IL-6 mRNA expression level in fresh stromal cells. (a) Splenic CD45^−^ stromal cells from young and aged mice were cultured with 0, 1, and 10 *μ*g/mL LPS for 24 hours, and IL-6 in the supernatant was measured by ELISA. *P < 0.05. The data are the representative of two similar experiments (*n* = 6). (b) IL-6 mRNA of freshly isolated splenic CD45^−^ stromal cells from young and aged mice (*n* = 3) was measured by quantitative RT-PCR. The results are expressed as mean ± SD. The average normalized IL-6 mRNA level in aged mice was set at 1.0. *P < 0.05.

To identify cells that highly produce IL-6 protein with aging, we next focused on stromal cells. We isolated CD45^−^ stromal cells from young and aged mice spleen by MACS. FACS analysis was performed and the cell population was characterized as >98% for CD45^−^ stromal cells. The IL-6 protein production levels were high in aged mice compared to young mice in all tested LPS concentrations (0, 1, and 10 *μ*M) (Figure 2(a)). CD45^−^ stromal cells from young mice produced lower amounts of IL-6 protein than those from aged mice and they showed only modest changes in IL-6 protein production when stimulated by LPS. Contrary to young CD45^−^ stromal cells, aged CD45^−^ stromal cells yielded higher levels of IL-6 protein even in noninflammatory state, and by stimulating with LPS, IL-6 production further increased and the production was significantly high compared to those of young mice. We then extracted RNA from freshly isolated CD45^−^ stromal cells and mRNA levels were investigated. Expression of IL-6 mRNA was significantly increased in the aged CD45^−^ stromal cells (Figure 2(b)).

### 3.3. Cell Populations of Spleen Both of Young and Aged Mice Do Not Show Age-Dependent Differences but Those of Splenic CD45^−^ Stromal Cells Show Different Profile

**Figure 3:**
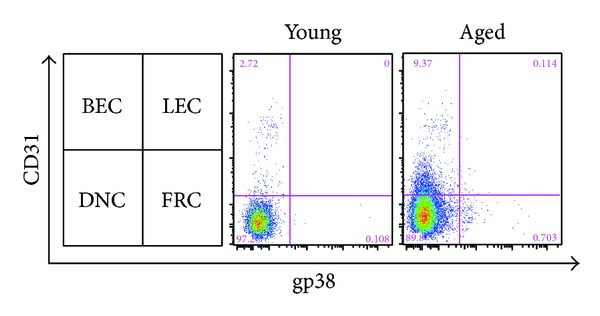
Splenic stromal cell populations of young and aged mice. SPL whole cells from young and aged mice were stained with anti-CD45, anti-CD31, and anti-gp38. Numbers in quadrants indicate percentage of each stromal cell subset among CD45^−^ cells. The data are the representative of three similar experiments (*n* = 3).

Cell populations might change with aging in spleen, and the difference in cell population may influence the overall production of IL-6 in the spleen of young and aged mice. The absolute cell number of spleen from aged mice was higher than young mice, which of young mice was 2.46 × 10^8^ ± 0.58 × 10^8^ and aged mice was 3.26 × 10^8^ ± 1.07 × 10^8^. To analyze the effect of population, we characterized the proportion of cells expressing F4/80^+^, CD11b^+^, CD11c^+^, CD4^+^, B220^+^, and CD45^−^ in spleen of young and aged mice by performing flow cytometric analysis. Aging process does not significantly affect cell population in the spleen and it was suggested that the difference in IL-6 production of splenocytes between young and aged mice was not due to the difference of the proportion of each cell population (Table 1). Flow cytometry analyses were performed to analyze distribution of populations within CD45^−^ stromal cells (Figure 3). Difference between young and aged mice was observed in BEC population of CD45^−^ stromal cells. CD45^−^ stromal cells of aged mice show high population of BEC and low population of DNC.

### 3.4. IL-6 Secretion of CD45^−^ Stromal Cells but Not CD45^+^ Cells Is Enhanced by Aging

**Figure 4:**
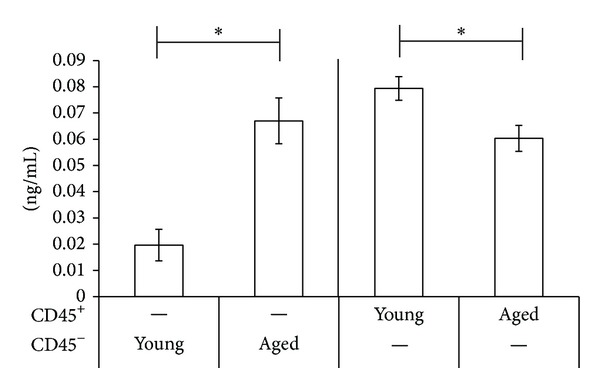
IL-6 protein induced by LPS in CD45^−^ or CD45^+^ cells. Spleen-derived CD45^−^ or CD45^+^ cells from young and aged mice (*n* = 4) were cultured with 10 *μ*g/mL LPS for 24 h. Supernatants were collected from the cell culture and were analyzed for IL-6 by ELISA. The results are expressed as mean ± SD. The data are the representative of two similar experiments.

To investigate further whether the enhanced IL-6 secretion of splenocytes from aged mice results from IL-6 production of aged CD45^−^ stromal cells, we cultured CD45^−^ stromal cells and CD45^+^ cells from aged and young mice and these cells were activated with LPS for 24 h. Five × 10^4^ of CD45^−^ stromal cells and 5 × 10^5^ of CD45^+^ cells from young and aged mice were incubated respectively and we confirmed that CD45^−^ stromal cells of aged mice secreted almost the same amount of IL-6 protein as ten times the number of CD45^+^ cells (Figure 4). When CD45^+^ cells were incubated, CD45^+^ cells from young mice secreted significantly higher IL-6 protein than those of aged mice. The results suggested that the higher IL-6 production from splenocytes of aged mice compared to young mice was due to the enhanced production of IL-6 in stromal cells of aged mice.

### 3.5. IL-6 Expression Was Elevated in Splenic Stromal Cells of Aged Mice after LPS Stimulation In Vivo

**Figure 5:**
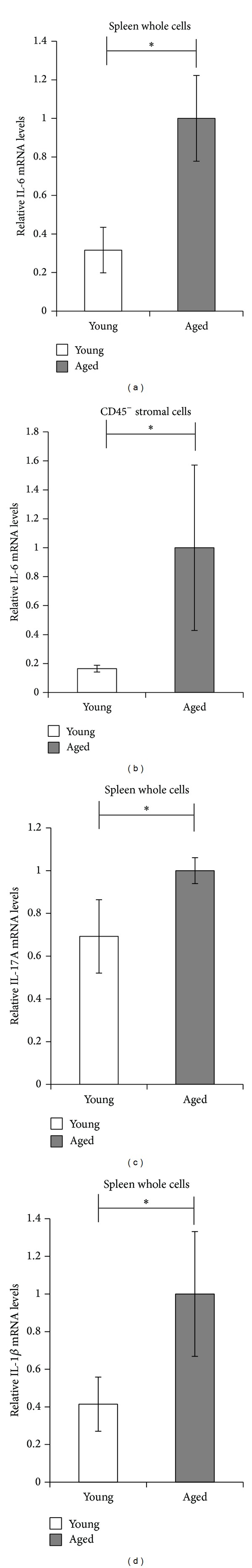
IL-6 and IL-17 mRNA expression levels in whole spleen cells and CD45^−^ stromal cells in vivo after LPS intraperitoneal administration for 1.5 h. (a) IL-6 mRNA of freshly isolated whole spleen cells from young and aged mice (*n* = 3) was measured by quantitative RT-PCR after LPS injection (3.3 *μ*g/g body weight). The average normalized IL-6 mRNA level in aged mice was set at 1.0. (b) IL-6 mRNA of splenic CD45^−^ stromal cells from young and aged mice (*n* = 5) sacrificed 1.5 h after injection was measured by quantitative RT-PCR. The average normalized IL-6 mRNA level in aged mice was set at 1.0. *P < 0.05. (c) IL-17 mRNA and (d) IL-1*β* of freshly isolated whole spleen cells from young and aged mice (*n* = 3) were measured by quantitative RT-PCR after LPS injection. The average normalized IL-17 mRNA level in aged mice was set at 1.0. The results are expressed as mean ± SD.

To investigate age-related overexpression of IL-6 after LPS stimulation in vivo, mice were injected i.p. with LPS, and IL-6 mRNA expression of splenocytes was assessed. We found that after LPS stimulation in vivo, aged mice spleen expressed significantly higher levels of IL-6 mRNA (Figure 5). To further elucidate whether CD45^−^ stromal cells of spleen exhibited age-related differences in IL-6 mRNA levels after LPS administration in vivo, spleens were obtained from young and aged mice after LPS injection, and stromal cells were isolated. As expected, CD45^−^ stromal cells of aged mice expressed significantly higher IL-6 mRNA after LPS stimulation in vivo (Figure 5). Since it is reported that IL-17 can enhance IL-6 production in concert with IL-6 in nonimmune cells, we also examined the expression of IL-17 mRNA [16]. The results demonstrated that IL-17 mRNA expression was also significantly higher in splenocytes of aged mice compared to young mice (Figure 5). In addition, it is reported that IL-1*β* also can induce higher level of IL-6 mRNA [26]; we investigated IL-1*β* mRNA expression. The results demonstrated that IL-1*β* mRNA expression was also significantly higher in splenocytes of aged mice compared to young mice (Figure 5).

## 4. Discussion

In this study, we examined the effect of aging process on IL-6 secretion, a proinflammatory cytokine, in several types of cells from spleen of young and aged mice. In autoimmune diseases, unbalance and dysregulation of proinflammatory cytokines are considered to play a pivotal role to develop autoimmunity [27]. Patients with elevated IL-6 show higher mortality and organ failure and anti-IL-6 receptor antibody is supposed to be effective medication for rheumatoid arthritis and Castleman's diseases [28]. It implies that IL-6 is a critical factor in autoimmune diseases, especially in rheumatoid arthritis [29–31]. There are a number of studies concerning cytokine secretions in aged mice and analyzing the age-related defects by showing changes of proinflammatory cytokines [7]. Aged mice have been shown to express increased levels of IL-6, including the serum IL-6 level after LPS treatment, and the results of our present study are consistent with these reports [3, 32–34].

In rheumatoid arthritis, it has been proposed that bacterial infections might play important roles, so the expression of proinflammatory cytokines in response to bacterial stimulation may also be important [35]. In this study, we observed that, upon LPS stimulation, splenocytes of aged mice secreted more IL-6 than those of young mice.

There are many cell types in the spleen including macrophages, dendritic cells, and other immune and nonimmune cells. We separated several types of cells from spleen and cultured these cells with or without LPS to identify cells that play roles in production of IL-6 with aging in inflammatory and noninflammatory environment with young and aged mice. Although macrophages produce IL-6 in the early phase of infectious inflammatory response [36], we found that the aging process does not affect the IL-6 production of these cells. Similarly, dendritic cells from both young and aged mice had almost similar ability to secrete IL-6. Rather, we found that the IL-6 production of CD45^−^ stromal cells was greatly increased in aged mice. We separated CD45^−^ stromal cells from young and aged mice spleen and cultured these cells with or without LPS for 24 h. Our results showed that CD45^−^ stromal cells of aged mice strongly produced IL-6 even under the noninflammatory condition, and the IL-6 protein production increased upon LPS stimulation (Figure 2). In addition, IL-6 mRNA was expressed highly in freshly isolated CD45^−^ stromal cells from aged mice compared to young mice, which alludes to the function of CD45^−^ stromal cells in secreting IL-6 changes with aging. To further investigate the effect of CD45^−^ stromal cells on IL-6 protein production of spleen upon LPS stimulation, we cultured CD45^+^ cells and CD45^−^ stromal cells under LPS stimulated condition for 24 h, respectively. CD45^+^ cells from young mice secreted higher IL-6 protein compared to aged mice, suggesting that CD45^+^ cells do not play a role in increased production of IL-6 with aging. This demonstrates that although hemopoietic cells other than dendritic cells may produce IL-6, the level of production did not differ between young and aged mice, and these cells did not contribute to the IL-6 secretion. And although the proportions of T cell subsets may have changed with aging, we consider that the effect on the production of IL-6 should be minimal. CD45^−^ stromal cells from aged mice secreted almost the same amount of IL-6 protein with CD45^+^ cells with one-tenth of CD45^+^ cells and CD45^−^ stromal cells from aged mice showed three times higher IL-6 protein yields than young mice. These results suggest that CD45^−^ stromal cells are crucial to increase the IL-6 protein production after LPS stimulation in aging process. CD45^−^ cell population can be distinguished into four groups depending on the expression of CD31 and Gp38 on the cell surface, BECs (blood endothelial cells, CD31^+^Gp38^−^), FRCs (fibroblastic reticular cells, CD31^−^Gp38^+^), LEC (lymphatic endothelial cells, CD31^+^Gp38^+^), and the double negative populations [20]. The distribution of subsets of these CD45^−^ stromal cells changed with aging (Figure 3). The BEC population increased in CD45^−^ stromal cells of aged mice. Although the ratio of CD45^−^ stromal cells did not differ, the composition of CD45^−^ stromal cells changed with aging. It implies that altered distribution of CD45^−^ stromal cells with aging, especially increased population of BECs, might contribute to augment IL-6 production.

IL-6 is chiefly regulated by NF-*κ*B [4]. We investigated NF-*κ*B p65 activation by western blotting using nuclear protein of splenocytes from young and aged mice. NF-*κ*B p65 protein content of nuclear extracts from aged mice was high compared to young mice (data not shown). It implies that, via activated NF-*κ*B p65, IL-6 is induced and results in a characteristic proinflammatory profile in aged mice. The molecular mechanism of IL-6 secretion from stromal cells is still not clarified, but this would require a novel approach and would be a subject of future study.

Recent data showed that IL-17 stimulates stromal cells to induce production of inflammatory mediators such as IL-6 in concert with IL-6, which indicates an IL-17-mediated IL-6 amplifying system [16, 37, 38]. Under the presence of IL-17, nonimmune cells including fibroblasts can secrete increased amounts of cytokines in response to infection. IL-17 treatment alone, however, is a poor stimulus for gene expression in these cells. Nevertheless, if IL-17 cooperates with other cytokines, especially TNF-*α* and IL-6, synergic response would occur and therefore it might be considered as a potential mechanism that in aged mice IL-6 production can increase significantly by IL-17A-triggered positive-feedback loop mediated by IL-17 and IL-6 [39]. We investigated IL-6 and IL-17A mRNA of young and aged mice spleen after 1.5 h LPS injection in vivo and our results show that splenocytes of aged mice highly expressed IL-6 and IL-17A mRNA than young mice spleen. Also, we confirmed that CD45^−^ stromal cells from aged mice expressed 5-fold higher IL-6 mRNA after 1.5 h LPS injection than from young mice. Our results imply that highly produced IL-6 by stromal cells and IL-17A in aged mice trigger the IL-6 amplifier and it results in increasing IL-6 production in aged mice spleen. It has been reported that IL-1*β* produced in LPS-injected mice stimulates cardiac cells to produce IL-6 [26]. We also found that expression of IL-1*β* was higher in splenocytes of aged mice compared to young mice after LPS injection. Yet another possibility may be that IL-1*β* produced upon LPS stimulation enhances IL-6 secretion in aged mice.

Our observations that increased IL-6 expression in splenic CD45^−^ stromal cells of aged mice suggest general etiological mechanisms of the chronic inflammatory condition found in the elderly, referred to as inflamm-aging. Such altered immune functions might have influence on developing diseases, susceptibility to infections, and conditions related to allergy in the elderly. In both of our in vivo and in vitro results, stromal cells play a role of causing difference in IL-6 production between young and aged mice. These results propose that increased risk with age of autoimmune diseases, especially induced by IL-6, is due partly to elevated IL-6 production by stromal cells. Additional studies are needed to make clear the mechanisms related to elevated secretion of IL-6 in stromal cells with age. And to better understand age-related IL-6 production in stromal cells, further studies concerning secretion of IL-6 in each subset of stromal cells might be necessary.

## 5. Conclusions

In conclusion, our results showed that, in the mouse spleen, the production of IL-6 was elevated with aging and that IL-6 production by stromal cells played a major role in this enhancement. Our results suggest the possibility that augmented production of proinflammatory cytokines such as IL-6 by stromal cells might be a cause of inflammatory diseases in the elderly.

## Figures and Tables

**Table 1:** The expression of surface markers on splenocytes of young and aged mice.

	Young	Aged
F4/80^+^	3.87 ± 1.59	3.57 ± 0.70
CD11b^+^	7.17 ± 2.63	5.87 ± 0.39
CD11c^+^	0.56 ± 0.13	0.78 ± 0.05
B220^+^	63.07 ± 2.26	61.08 ± 4.33
CD4^+^	17.15 ± 1.62	18.63 ± 2.95
CD45^−^	1.80 ± 2.35	2.81 ± 2.76

Splenocytes were obtained from young and aged mice (*n* = 4~5). Cells were stained for the surface markers F4/80, CD11b, CD11c, B220, CD4, and CD45. Values indicate the mean percentages of each subset. Data are expressed as the mean ± SD.

## Abbreviations

SLO: Secondary lymphoid organs.
